# Transobturator tapes are preferable over transvaginal tapes for the management of female stress urinary incontinence: Against

**DOI:** 10.4103/0970-1591.57903

**Published:** 2009

**Authors:** Arun Chawla

**Affiliations:** Department of Urology, Kasturba Medical College, Manipal 576 104, Karnataka, India

**Keywords:** Stress urinary incontinence, transvaginal tape, transobturator tapes

## Abstract

Midurethral placement of tension-free vaginal tapes with a transvaginal route for stress urinary incontinence achieves higher and better long-term success rates than the transobturator route. Bladder perforations are reported more in transvaginal tape (TVT) but incidences of vaginal erosions, extrusion, and groin pain are exceedingly more in TOT groups. There is no clear evidence that transobturator tape (TOT) is associated with less post-operative voiding problems than TVT. Major complications such as bowel injuries and significant vascular injuries with TVT are rare. TVT has been found to be superior to TOT and preferable in technically demanding conditions such as prior anti-incontinence operation failures, obese women, and very elevated and scarred lateral cul-de sac. TVT is always preferred in severe grades of stress urinary incontinence and with patients of intrinsic sphincter deficiency (ISD) with little or no urethral mobility.

Stress urinary incontinence (SUI) in females can be treated by transobturator (TOT) and transvaginal (TVT) placement of mid-urethral tapes. Are transobturator tapes preferable over transvaginal tapes for the management of female stress urinary incontinence? The answer to this question can be found by discussing the following issues.

- Which mid-urethral tape has the maximum efficacy and documented favorable long-term results?
- Which type of mid-urethral tape is associated with overall increased complication rates?
- Are the results in both the mid-urethral tapes the same for severe grade of SUI and intrinsic sphincter deficiency?
- Which mid-urethral tape is more useful in complex and difficult situations?

## EFFICACY AND LONG-TERM RESULTS

The TVT procedure for the treatment of female stress incontinence is the first modern minimally invasive mid-urethral sling that was started in 1996. The vast majority of published literature regarding midurethral tapes relates to TVT procedures. Two randomized trials of TVT *vs*. open Burch Colposuspension (considered to be the gold standard for the treatment of SUI) revealed no difference in the objective and subjective cure rates.[1] Reported rates of short-term and long-term success rates are definitely higher in TVT as compared with the TOT group. A large cohort analysis and long-term prospective evaluation indicated a cure rate of 81% and an improvement rate of 94% in TVT.[2] TOT was effective in only 83% of the patients and TVT was effective in 100% of the patients at the 1-year follow-up visit.[3] Lathe, *et al*.[4] in his meta-analysis of various randomized trials using TVT and TOT procedures observed that the subjective cure rate of SUI was worse in the TOT group as compared with the TVT group. Any treatment of SUI should be successful in the cure of incontinence and should stay effective for the long-term. The results of the long-term prospective follow-up of the efficacy of the TVT procedure are reassuring and no distinct decline in cure rates occurred over a period of 11 years.[2] The TOT procedure was started in 2001 and long-term data on successful outcomes in the TOT group is lacking.

## COMPLICATIONS

### Bladder injuries

The most common complication of TVT is bladder perforation and is reported to occur in 0.7% to 24% of cases.[5] The risk factors include a history of anti-incontinence surgery, pelvic surgery, and surgeon experience. The incidence seems to decrease as the experience with the procedure increases. Careful and circumferential cystoscopic examination of a distended bladder can easily obviate this complication. If perforation is detected, the trocar is removed and re-passed and the cystoscopy is repeated with each pass of the trocar. In difficult situations, injection of normal saline behind the pubic bone in the intended path of needles or passing the needle from the suprapubic incision to the vaginal tunnel or small laparotomy to pass the trocar under direct vision can be attempted. Although, bladder and urethral injuries are uncommon in TOT procedures, the incidence is not zero. In the Austrian TOT tape registry comprising of 2,541 TOT surgeries, 10 bladder and 2 urethral perforations are reported.[6] In a retrospective study of 390 patients treated with TOT, 2 urethral and 2 bladder injuries were reported.[7] Minaglia, *et al*.[8] recommend universal intra-operative cystoscopy when performing TOT especially in previous extensive pelvic surgery and when needle passage is difficult.

### Hemorrhage

Hemorrhagic complications can be associated with the passage of any needles placed retropubically or via the transobturator route. The vascular situation in the Retzius space is unknown as is the adhesion of the bladder to the pubis. Retro pubic hematoma and bladder perforation may happen in TVT procedures. This risk is high in patients with previous surgery in the Retzius space. In TVT, the reported incidence of increased blood loss ranges from 1.1% to 2.3%, while the rate of retropubic hematoma ranges from 2.0% to 4.1%.[6] Mean distance from the trocar to the major vessels is 3.2 to 4.9 cm and vascular injuries involving large arteries such as the external iliac femoral, obturator, epigastric and inferior vesical, though reported in few reports, are rare. Minor bleeding in the retropubic procedure may relate to the close positioning of the dorsal vein of the clitoris under the inferomedial aspect of the pubic bone. Hemorrhage related complications do occur in the TOT procedure in 1–2% of the cases and includes heavy intaoperative bleeding, pelvic hematoma, retropubic hematoma, and perineal, labial or thigh hematoma.[6]

### Vaginal Erosion and Urethral Erosion

Vaginal erosions are uncommon in patients treated with TVT procedures. The subgroup analysis of treatment with TVT and TOT groups showed that the majority of vaginal perforations occurred in the TOT group. Vaginal erosions are probably the result of inadvertent, unrecognized vaginal wall punctures during a TOT procedure and contributing factors are tension between the mesh and vaginal epithelium, inadequate reapproximation of vaginal tissues, sub-clinical infections of sling material, and impaired vascularity. Deval[9] reported incidence rates of vaginal erosions in TVT and TOT tapes as 0.7% and 13.8%, respectively. Lathe, *et al*.[5] in a systematic review and meta-analysis of the effectiveness and complications following both the procedures reported the incidence of vaginal injuries, erosion, and extrusion twice in the TOT group as compared with the TVT group. Vesicovaginal fistulas have also been reported after TOT surgeries. Mesh erosion into the urethra though rare in both tapes can occur due to poor dissection or excessive tension on the tape. Neelson, *et al*.[2] in their follow-up of TVT patients observed no case of tape erosion, tissue reaction, or other adverse effects of the tape up to the 13 years of follow-up. Recently, high rates of vaginal erosion have been seen in TOT procedures using URA Tape/Ob Tape.[9] Seigel, *et al*.[10] reported a 20% vaginal extrusion in patients with Ob Tape (TOT tape). Take down rates following TOT and TVT procedures ranges from 2.8% to 3.9% and 1. 7% to 2.8% have been reported.[11,12]

### Bowel perforation

Reports of bowel perforation in the TVT group in published data over the last 10 years are rare. Patients with a history of previous abdominal or pelvic surgery are at a greater risk because of adhesion in the Retzius space. It has been suggested that an MRI or CT may be useful in preoperative planning in these patients. A small laparatomy so that trocars can be passed under direct vision is more judicious in these circumstances. No bowel or vascular injuries have been reported by David Montefire, *et al*.[13] in the randomized study comparing perioperative complications in the TVT and TOT groups.

### Denovo Urgency and Urge Incontinence

Denovo urgency after TVT ranges from 6.3% to 15% and is thought to result from a combination of a degree of urethral obstruction and urethral irritation from the presence of the sling.[6] This probably is linked to changes in paraurethral collagen metabolism and sclerosis around the tape. In the meta-analysis of TVT and TOT procedures. Lathe, *et al*.[5] observed that denovo frequency and urgency symptoms were equivalent in both the groups. Ballert, *et al*.[14] reported that denovo urgency and urge incontinence rates range from 0.2% to 15% after TVT and 2.1% to 13.9% after placement of TOT mid-urethral slings. Pre-existing urge symptoms have been shown to resolve in many patients with a TVT procedure. Sinha, *et al*.[15] in their study of 40 women with mixed urinary incontinence found that a majority had a complete cure or an improvement in urge symptoms along with improved or cured stress incontinence following placement of the TVT, which is contrary to the belief that TVT procedures may have contributed to the deterioration of symptoms. Ulmstein[16] found an 85% objective cure rate and 90% improvement in quality of life in 80 women undergoing TVT procedures for mixed urinary incontinence.

### Voiding dysfunction

Reported rates of voiding dysfunction after TVT procedures range from 2.5% to 12.6% although analysis of results is difficult because of an inconsistent definition of this voiding dysfunction.[17] Obstructive voiding symptoms using midurethral tapes are influenced by more than just the approach. The highest obstruction inducing risk is the surgeon himself tensioning the tape (it's supposed to be tension free) too much. In a patient with immediate post-operative retention or incomplete bladder emptying, an indwelling catheter or intermittent self catheterization is tried as a spontaneous resolution is common. A simple sling lysis is considered in a persistent voiding problem. Obstructive voiding has been reported to occur in 1.5% to 15.6% of patients after a TOT procedure while in a TVT procedure it ranges from 7.8% to 11.2 %.[17] A very low rate of post-operative voiding problems without any change in the maximum flow rate in both the groups has been reported.[18] Costa, *et al*.[19] and Dawson, *et al*.[17] reported voiding difficulties of 5.4% and 3.8%, respectively that needed self catheterization. The predictable variables for this complication after TVT procedures are abnormal uroflow pattern (maximum flow rate < 15 ml /sec) preoperative enterocele or vault prolapse, combined vault suspension with TVT, and an increased age of the patient.[17]

### Groin Pain and Abscesses

Persistent groin pain after a TVT procedure is very uncommon. Laurikeuen, *et al*.[11] in his comparison of 136 patients in the TVT group with 131 patients in the TOT group observed that groin pain is significantly more in TOT patients than in TVT patients (16% *vs*. 1.5%) and considerably more opiate analgesia (21% Vs 12%) was needed in the TOT group.[14] The possible explanation is a path of insertion through the mascular or tendineous portion of the adductor muscles. Compression or entrapment of the anterior obturator nerve during the TOT procedure may also add to groin pain or exercise related pain. If this does not respond to steroids, analgesics or local injection of anesthetic, dissection, and excision of distal end tape is needed. Butt, *et al*.[20] in their study of 2 different obturator techniques found that pain duration varied from 5-7 days (outside–in) to 6–21 days (inside–out) procedures significantly affecting quality of life. Four neuropathies resulting in gait disturbance in TOT has also been reported in the MAUDE (Manufacturer and User Facility Device Experience) database. The MAUDE database lists 25 infection-related complications in TOT groups, such as ischiorectal and adductor muscle abscesses, including one death. Such complications are rare in TVT procedures.

Both tapes have different complications inherent to their route of insertion. Major complications related to injury of bowel and major blood vessels with placement of TVT are rare. Many recent reports suggest very low incidence of bladder perforations in TVT and incidence of postoperative voiding problems are similar in both the groups. Vaginal extrusion and erosions are significantly higher in the TOT group. Persistent groin pain, more commonly seen after a TOT procedure, is bothersome and can significantly affect a patient's quality of life.

### Severe Incontinence with Intrinsic Sphincter Deficiency

The TVT procedure has shown more efficacy than the TOT procedure in curing patients with severe incontinence and success rates in patients with ISD is higher in the TVT procedure than the TOT procedure. Araco, *et al*.[21] in his large randomized study of TVT and TVT-O for SUI observed that all patients insufficiently cured were those affected by severe stress incontinence and treated by TVT-O. In their study,[17] patients who failed with TVT-O underwent a TVT procedure with successful outcomes. The authors believe that the TVT procedure is more obstructive and that patients with severe incontinence could benefit more from a TVT procedure than from a TOT procedure. Satisfactory outcomes in patients with SUI and ISD treated with TVT has been reported.[22] A alsalva leak point pressure (VLPP) less than 60 cm H_2_O did not predict the failure after a TVT procedure and patients with SUI, adequate or minimal urethral mobility, and low micturating urethral closure pressure (MUCP) could expect a very significant success rate after the TVT procedure.[23] Similar data on the use of TOT is lacking as this procedure is never used in these circumstances.

## COMPLEX SITUATIONS

Literature advocates the use of a TVT procedure not only in pure SUI patients but also in those having a simultaneous prolapse repair and has better success rates in patients presenting with recurrent or persistent SUI.[12] A very elevated and scarred lateral cul-de sac makes the passage of the TOT helix impossible and therefore unsuitable for TOT procedures. The TVT procedure has been shown to be effective in obese women and women where prior anti-incontinence surgery has failed.[24] Similar data on the TOT procedure is not available.

In conclusion, the TVT procedure achieves higher and better long-term success rates in the treatment of SUI than the TOT procedure. Each procedure has its own set of complications. In uncomplicated patients with mild to moderate stress or mixed incontinence, both procedures are equally safe. The TVT procedure is much more effective in prior anti-incontinence operation failures, obese women, and very elevated and scarred lateral cul-de sac and is preferable than the TOT procedure. The TVT procedure is always preferred over TOT in severe grades of stress incontinence and with patients having ISD with little or no urethral mobility.
